# Interpretable Machine Learning Techniques in ECG-Based Heart Disease Classification: A Systematic Review

**DOI:** 10.3390/diagnostics13010111

**Published:** 2022-12-29

**Authors:** Yehualashet Megersa Ayano, Friedhelm Schwenker, Bisrat Derebssa Dufera, Taye Girma Debelee

**Affiliations:** 1 Addis Ababa Institute of Technology, Addis Ababa University, Addis Ababa 11760, Ethiopia; 2 Institute of Neural Information, University of Ulm, 89069 Ulm, Germany; 3 Ethiopian Artificial Intelligence Institute, Addis Ababa 40782, Ethiopia; 4 College of Electrical and Computer Engineering, Addis Ababa Science and Technology University, Addis Ababa 16417, Ethiopia

**Keywords:** interpretable, machine learning, IML, ECG, heart disease

## Abstract

Heart disease is one of the leading causes of mortality throughout the world. Among the different heart diagnosis techniques, an electrocardiogram (ECG) is the least expensive non-invasive procedure. However, the following are challenges: the scarcity of medical experts, the complexity of ECG interpretations, the manifestation similarities of heart disease in ECG signals, and heart disease comorbidity. Machine learning algorithms are viable alternatives to the traditional diagnoses of heart disease from ECG signals. However, the black box nature of complex machine learning algorithms and the difficulty in explaining a model’s outcomes are obstacles for medical practitioners in having confidence in machine learning models. This observation paves the way for interpretable machine learning (IML) models as diagnostic tools that can build a physician’s trust and provide evidence-based diagnoses. Therefore, in this systematic literature review, we studied and analyzed the research landscape in interpretable machine learning techniques by focusing on heart disease diagnosis from an ECG signal. In this regard, the contribution of our work is manifold; first, we present an elaborate discussion on interpretable machine learning techniques. In addition, we identify and characterize ECG signal recording datasets that are readily available for machine learning-based tasks. Furthermore, we identify the progress that has been achieved in ECG signal interpretation using IML techniques. Finally, we discuss the limitations and challenges of IML techniques in interpreting ECG signals.

## 1. Introduction

Heart disease is one of the deadliest health conditions affecting the heart and blood vessels. According to a World Health Organization (WHO) report, in the year 2019, around 17.9 million cardiovascular disease-related deaths were registered. This accounts for 32% of all global mortality, and the highest among all non-communicable diseases [1]. In addition, more than three-fourths of all these mortalities occur in low and middle-income countries [1].

Clinicians diagnose heart disease via different techniques, including non-invasive methods, such as an electrocardiogram (ECG) [2], echocardiogram [3], coronary computed tomography angiogram (CCTA) [4], cardiac magnetic resonance imaging (MRI) [5], and invasive techniques, such as blood tests [6] and coronary angiograms [7]. Among the listed diagnosis techniques, ECG is a low-cost and non-invasive procedure that can easily be administered for diagnosing heart disease [2]. Thus, an ECG-based diagnosis is used for detecting and diagnosing various heart diseases, such as arrhythmia, pericardia, myocardia, electrolyte disturbances, and pulmonary diseases [2,8]. However, physicians at all levels experience difficulties in accurately interpreting ECGs [9]. J. Higueras et al. [10] reported that from a study group of 195 physicians (where 153 of them were residents and 42 staff) that ECG interpretation skills among medical doctors are poor. According to the study, heart disease, such as acute myocardial infarction (AMI), ventricular tachycardia (VT), and a second degree AV block missed with 13.4 %, 44.1%, and 64.6% by the resident physicians, respectively. In addition, the existence of different types of heart disease conditions poses a challenge for making a diagnosis through reading an ECG signal, even by a well-trained cardiologist. Moreover, the similarities of heart disease manifestations on ECG signals pose extra challenges for properly distinguishing them. Apart from these challenges, the ECG signal recording may show discrepancies for the same disease condition based on age, race, and the overall physical conditions of patients [2].

To mitigate these challenges and aid physicians in the diagnosis of heart conditions, a computerized interpretation of ECG records (CIE) was introduced [11]. However, studies have shown significant inaccuracies of this method and limitations of computerized ECG interpretation [12]. Thus, despite attempts to improve the accuracies of automated ECG interpretation techniques, the final ECG interpretation still requires a physician re-read. Furthermore, the lack of an internationally accepted standard for computerized ECG interpretation poses a challenge to relying on CIE [11].

### 1.1. ECG Signal

**Figure 1:**
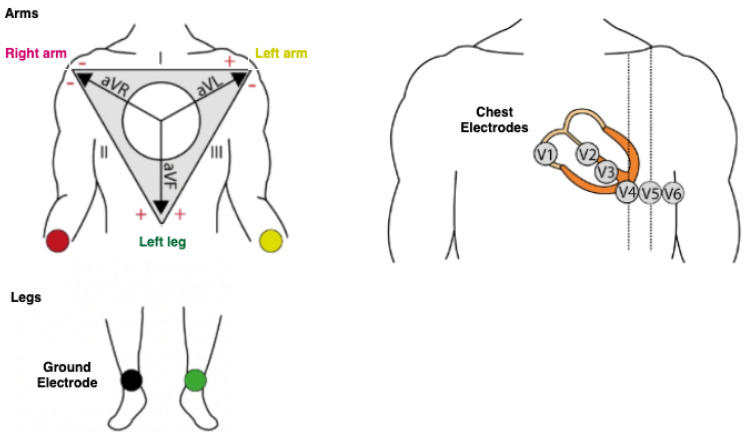
The placement of ECG electrodes on the chest, arms, and legs [16].

**Figure 2:**
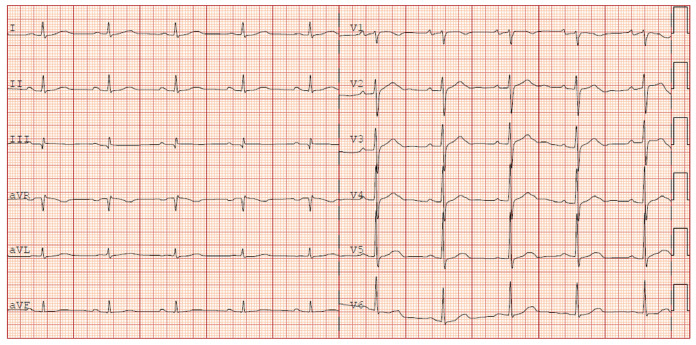
A standard 12-lead ECG of a single patient [15].

ECG machines are used for the acquisition of electrical activities of the heart as observed from the sensors/electrodes attached to a patient’s arms, legs, and chest, as shown in Figure 1. The electrical signals picked by these electrodes are associated with a 12-lead ECG machine that records the aggregate electrical activity of the heart from distinct angles over some time, commonly 12 s [13]. Among the 12-leads, the three bipolar leads measure the potential differences between both arms, and one arm and the leg [14]. The remaining nine electrodes are unipolar and consist of six chest leads (V1 to V6), which view the heart in the horizontal plane, and six limb leads (I, II, III, aVR, aVL, and aVF), which help to view the heart in the vertical plane [2,15], as shown in Figure 1. A standard ECG record of a patient is shown in Figure 2.

**Figure 3:**
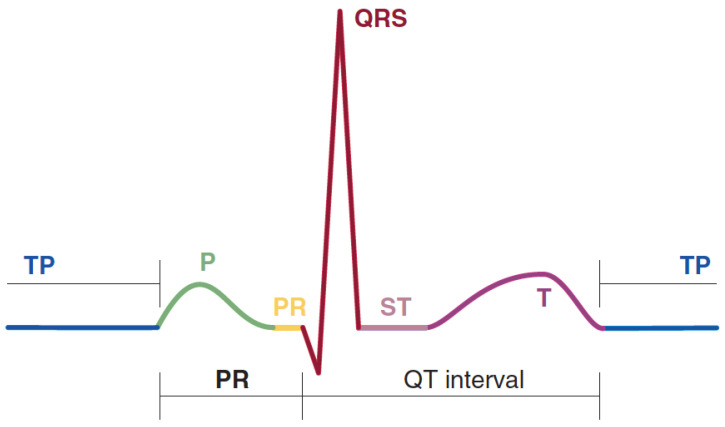
A single cardiac cycle of the ECG pattern [14].

A single cycle of an ECG contains a pattern of waves, as shown in Figure 3. When the sinoatrial (SA) node triggers an impulse, the atrial fibers depolarize to produce a potential difference called a *P wave*, leading to atrial contraction. In a normal ECG, as shown in Figure 3, a *P wave* has a duration of about 0.08 s [14]. A *P wave* is seen in leads II and V1. Moreover, it leans inverted in the lead aVR and is upright in leads I and II, as shown in Figure 2.

After the atrial fiber depolarization, the impulse reaches the ventricular fibers and rapidly depolarizes them. Since the ventricular walls are thick, the depolarization results in more electrical changes; it is called the *QRS complex*, which consists of Q, R, and S waves. The *QRS complex* also lasts for about 0.08 s [14]. Then, as the ventricles repolarize, a *T wave* is produced. The *T wave* is about 0.16 s in a normal ECG. It can be seen from Figure 3 that the atrial repolarization is missing from the pattern due to atrial fiber repolarization at the same time as ventricular fiber depolarization [14].

**Figure 4:**
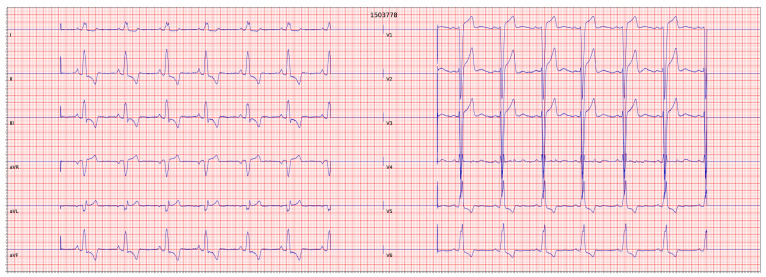
A 12-lead ECG of a patient with exam_id of 1503778 diagnosed for LBBB [18].

As shown in Figure 3, the PR interval is the period between the *P wave* and the *QRS complex*. The PR interval indicates the impulse transmission times between the SA and atrioventricular (AV) nodes. It contains atrial depolarization, contraction, and depolarization waves via the conduction system. The ST segment, on the other hand, occurs during the depolarization of the ventricular myocardium, and it lasts about 0.22 s. The QT interval that lasts about 0.38 s is a period from the start of ventricular depolarization to repolarization [14]. The TP segment is an isoelectric region that indicates the absence of a substantial amount of potential difference in the ventricular myocardial cells. It is a resting state of the ventricular myocardial cell and covers a time from the end of repolarization to the onset of the next depolarization [17]. Any deviation from this normal cardiac cycle may indicate heart disease and conduction system problems. As shown in Figure 4, for instance, a QRS duration greater than 0.12 s, broad monophasic R waves in leads I, V5, and V6, and the absence of Q waves in leads V5 and V6 are indications of the left bundle branch block (LBBB) [2].

### 1.2. Machine Learning: In an ECG Signal Classification Prescriptive

Recently, several studies have examined the possibility of artificial intelligence (AI) techniques in interpreting an ECG in the diagnosis of cardiovascular diseases [18,19,20,21,22,23,24,25,26,27,28]. In addition, a review article written by Liu et al. [29] provided a detailed review of deep learning techniques used for ECG diagnosis. Some of the literature examined AI-enabled techniques to classify up to 66 multi-label heart abnormalities using 12-lead ECG readings and reported promising results [30]. However, most of the literary studies focus on identifying small types of heart abnormalities from among several types of heart disease [18,31]. Moreover, some of the literary studies only focus on normal and abnormal ECG signal classes from a single lead ECG signal [23,26]. ML-based heart disease detection and classification methods from an ECG signal bring promising results and are active research areas. Some of the reported results demonstrate that the performances of ML-based ECG interpretation algorithms are better at approximating human experts compared to existing CIE techniques [30].

However, the difficulty of a machine learning (ML) model’s interpretability has hindered medical practitioners from having confidence in the diagnosis results of machine learning models [32]. ML model interpretation techniques provide evidence for a particular model’s output [32]. Moreover, these interpretation techniques enable human experts to trust the model’s output, debug and troubleshoot the model, and avoid model bias [33]. However, the field of explainable AI is not mature, and researchers are focusing on introducing techniques that can provide the reasoning of the model behind a particular detection or classification of abnormalities in healthcare settings [32] and other applications [33]. In this systematic review work, IML techniques that were proposed in the literature to give evidence-based ECG signal interpretations are discussed. Moreover, their performances are presented in terms of qualitative and quantitative approaches. In addition, this work focuses on pinpointing the strengths and limitations of the IML techniques in terms of computational complexity and result presentation.

The remainder of the paper is organized as follows: Section 2 discusses the recent related works to this systematic review work, and Section 3 elaborates on the techniques used to conduct the review and research the questions addressed in this review work. The most prominent (in terms of data size and disease class), i.e., annotated heart disease ECG data repositories, are discussed in Section 4. IML techniques proposed in the literature for explaining the ML model output developed for ECG signal-based heart disease classification are investigated and presented in Section 5. Section 6 discusses the performance evaluation methods for IML techniques focusing on ECG signal-based heart disease classification. The findings of this review work and existing challenges and future directions are discussed in Section 7 and Section 8. Finally, Section 9 presents the conclusion.

## 2. Related Work

This section discusses the related systematic review works to examine state-of-the-art research and challenges toward heart disease classification using interpretable machine learning (IML)-based techniques from ECG signal. To the best of our knowledge, systematic reviews that are related to IML-based heart disease classification from ECG signals are very limited in number and scope. However, some works have investigated and discussed the IML techniques from the point of view of healthcare applications, as well as the existing challenges and future directions in the field of medicine [32,34,35,36,37,38,39,40,41].

Abdullah et al. [32] provided a comprehensive survey on the uses of IML techniques in healthcare. The paper presented an in-depth theoretical discussion of the existing well-known IML techniques. However, only a single piece of literature was reviewed that focuses on the application of IML on ECG signal-based heart disease classification. Similarly, Rasheed et al. [36] reviewed a single literature study on IML-based ECG signal interpretation. However, they provide a comprehensive review of IML techniques that explain the reason behind their decisions. Likewise, Yang et al. [37], Stiglic et al. [38], and Jin et al. [41] did not provide reviews on the progress of interpretable techniques on ECG signal-based heart disease diagnosis. Instead, they described the progress made in using interpretable techniques in explaining black box ML models developed in different healthcare solutions. In addition, Yang et al. [37] showcased the benefits of ML model interpretable methods in explaining multi-modal and multi-fusion medical image segmentation. On the other hand, Stiglic et al. [38] emphasized feature importance-based ML model explanations. Whereas, Jin et al. [41] provided a discussion on the benefits and limitations of various ML model interpretability techniques to acquaint researchers and practitioners with IML in the fields of ML and medicine so that they can contribute to the field. However, the mathematical foundations in ML interpretable methods are not briefly discussed in these review works [36,37,38,41].

Du et al. [39] and Carvalho et al. [40] presented the need that necessitates explaining the prediction of complex ML models by providing human-friendly explanations within societal ethics and legal framework. In this regard, Du et al. [39] discussed some IML techniques and their categorization. Moreover, they outlined challenges to be addressed while designing and evaluating these techniques. Similarly, Carvalho et al. [40] provided an elaborated discussion on the categorization of IML techniques and presented the need for explaining ML by focusing on the societal impacts. In addition, the literature focused on identifying the mechanism for assessing the quality of the explanation and metrics to evaluate the explanations provided by IML techniques.

Xiong et al. [34] reviewed the most popular deep learning algorithms for detecting and locating myocardial infractions. Furthermore, the paper discussed the necessity of the model’s explainability for evidence-based medical diagnosis. However, the review did not include a discussion on IML-based myocardial infraction detection techniques. Similarly, Somani et al. [35], reviewed deep learning-based literature aimed at detecting and classifying five (5) types of heart disease from an ECG, including arrhythmia, cardiomyopathy, myocardial ischemia, valvulopathy, and non-cardiac diseases. The article pinpointed the potential of deep learning models in heart disease detection, especially for mass screening purposes. However, a very limited and shallow discussion on the interpretable model was presented. A summary of related works is given in Table 1.

## 3. Method

This section presents the methodology employed for reviewing the use of IML techniques for the detection of heart disease using an ECG signal. To that end, the preferred reporting items for systematic reviews and meta-analyses (PRISMA) [42,43] reporting technique is used to define the research questions, data sources (databases), and search strings for this particular research study. Based on the PRISMA guideline, the following steps are followed to accomplish our systematic review work.

- Defining the research questions;
- Based on the research questions, retrieving some keywords to create proper search strings;
- Identifying the databases for performing the search using the created search strings;
- Setting filtering criteria, including the chronological period, the quality, and the type of literature to be included in the review;
- Skimming titles and abstracts to avoid unrelated articles and duplicates from the pool of papers;
- Defining more detailed suitability criteria and using them in a full paper reading of the outlived papers from the previous steps;
- Analyzing and interpreting the outlived articles from all the filtering procedures in line with research questions defined in the beginning;
- Reporting and evaluating the systematic review.

### 3.1. Research Question

In synthesizing the empirical evidence for this systematic research work, four review questions are coined with their rationale as shown in Table 2.

### 3.2. Search Strategy

The database and search strings are selected in a way to address the research questions indicated in Table 2. The search focused on identifying the literature from the following seven main databases:

1. Google Scholar, a scholarly literature search engine that encompasses a wide variety of disciplines and publisher databases;
2. PubMed, a database consisted of a large number of literary studies in the biomedical field, primarily from the MEDLINE database;
3. IEEE Xplore, this database contains high-quality technical literature in the fields of electrical engineering, electronics, computer science, and other related fields;
4. ScienceDirect, using this database, access to journals and technical articles published by Elsevier is possible;
5. MDPI, a publisher of open-access peer-reviewed scientific journals;
6. Wiley Online Library, this is a repository of published articles in various disciplines, including computational, intelligent systems, and life sciences;
7. SpringerLink, through this database, we can access scientific articles published by Springer Nature.

By rigorously following the steps listed above, our systematic review work is aimed at achieving three targets: (1) to be used as a reference in the existing IML techniques that use ECG signals for heart disease classification; (2) to help researchers in avoiding work redundancy; (3) to aid researchers in the area to identify research gaps in an evidence-based heart disease diagnosis using IML.

To meet these targets, primarily, an elaborate discussion on interpretable machine-learning techniques will be presented. In addition, it identifies and characterizes heart disease ECG signal datasets that are readily available for machine learning-based research. Furthermore, it identifies the progress that has been achieved in ECG signal interpretation using IML techniques in terms of different IML model performance measuring techniques. Finally, it discusses the limitations and challenges of IML techniques in interpreting an ECG signal.

Search strings used to find the literature for this review work are tailored toward these seven databases to specifically focus on not missing literature from each of them. As a result, the search strings used for Google Scholar, ScienceDirect, PubMed, Wiley Online Library, and SpringerLink are the following: [(“Explainable” OR “Interpretable”) AND (“Machine learning Techniques” OR “Deep Learning Techniques”) AND (“Heart Disease”) AND (“Electrocardiogram” OR “ECG”) AND (“Detection” OR “Classification”)], for IEEE Xplore is: [(“All Metadata”: Interpretable) AND (“All Metadata”: Machine learning techniques) OR (“All Metadata”: Deep learning techniques) AND (“All Metadata”: Heart disease detection) AND (“All Metadata”: ECG signal)], and for MDPI is: [(“Interpretable OR Explainable”) AND (“Machine learning” OR “Deep learning”) AND (“Heart disease”) AND (“CG signal”)].

**Figure 5:**
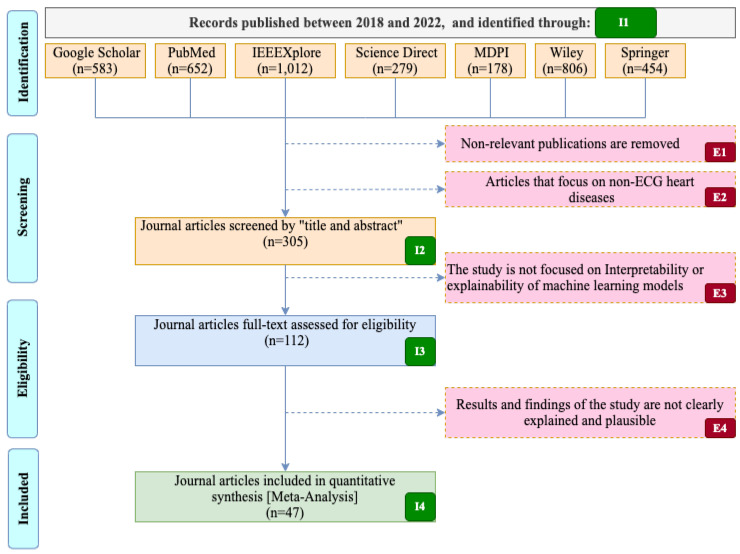
Flow diagram of paper selection.

The inclusion and exclusion criteria for the identified literature are indicated in Table 3. On the other hand, Figure 5 shows the literature selection process for our systematic review. Furthermore, the total number of journal articles identified for the quantitative analysis, and the stages for the inclusion and exclusion criteria used in the selection process are clearly shown in Figure 5.

## 4. Heart Electrocardiogram Diagnosis Datasets

In an ECG signal-based heart disease classification, several datasets exist and have been used to train and test ML models. However, these datasets differ in various ways, including sampling frequency, number of recording leads, and number of disease conditions or classes. The most prominent heart ECG datasets (in terms of data and disease class size) with their characteristics are given in Table 4.

The 2020 PhysioNet challenge dataset is compiled from five multiple data sources, which are the China physiological signal challenge [44], St. Petersburg INCART 12-lead arrhythmia database [45], PTB-XL ECG dataset [46], Georgia 12-lead ECG challenge [47], and undisclosed sources [47]. Other dataset repositories, such as MIT-BIH arrhythmia database [48], MIT-BIH atrial fibrillation database [49], MIT-BIH normal sinus rhythm database [50], BIDMC congestive heart failure database [51], normal sinus rhythm RR interval database [52], and many more have also been used to test different IML techniques. However, their data size are very few and provide beat- and -rhythm level annotations, as given in Table 5.

Except for the CODE dataset [18], the remaining data sources indicated in Table 4 are publicly available through their respective URLs. The CODE dataset is not public, although it can be obtained by signing data usage agreements with authors. However, 15% of the dataset is publicly available through the URL-indicated Table 4.

**Table 4:** Heart ECG signal diagnosis datasets.

Dataset	# of Lead	# of Records	# of Classes [Including Normal]	Samp. Freq. (Hz)	Website URL ^1^
Hannun et al. [53]	Single lead	91,232	12	200	https://irhythm.github.io/cardiol_test_set/ ^2^
2017 PhysioNet Challenge [54,55]	Single lead	8528	4	300	https://archive.physionet.org/physiobank/database/challenge/2017/
2020 PhysioNet Challenge [47,56]	12-lead	43,101	111	257, 500	https://physionet.org/content/challenge-2020/1.0.2/
Chapman University, Shaoxing People’s Hospital [57]	12-lead	10,646	11	500	https://physionet.org/content/ecg-arrhythmia/1.0.0/#files-panel
China Physiological Signal Challenge [44]	12-lead	6877	9	500	http://2018.icbeb.org/Challenge.html
PTB-XL ECG dataset [46,58]	12-lead	21,837	71	500	https://physionet.org/content/ptb-xl/1.0.1/
Shandong Provincial Hospital [59]	12-lead	25,770	44	500	https://springernature.figshare.com/collections/A_large-scale_multi-label_12-lead_electrocardiogram_database_with_standardized_diagnostic_statements/5779802/1
CODE dataset [18]	12-lead	2,322,513	7	300–600	https://zenodo.org/record/4916206#.Y1eIWuxBxmo ^3^

^1^ All website URLs were accessed on 25 October 2022. ^2^ Only test data are available through this URL. The complete dataset can be obtained upon request from Hannun et al. [53]. ^3^ Only 15% is available through this URL. The complete dataset can be obtained upon requesting from Ribeiro et al. [18].

**Table 5:** Beat, rhythm, and signal quality level of the annotated heart ECG signal datasets.

Dataset	# of Lead	# of Records	Annotation Type	# of Classes [Including Normal]	Samp. Freq. (Hz)	Website URL ^1^
MIT-BIH Arrhythmia database [48]	2 leads	48 two-channel half-hour recordings	Beat Rhythm Signal quality	20 classes of arrhythmia beats 15 classes of arrhythmia rhythms 5 classes of signal quality	360	https://physionet.org/content/mitdb/1.0.0/
MIT-BIH Atrial Fibrillation Database [49]	2 leads	25 two-channel 10-h recordings	Rhythm	4 classes of rhythms	250	https://physionet.org/content/afdb/1.0.0/
MIT-BIH Normal Sinus Rhythm Database [50]	2 leads	18 two-channel 24-h recordings	Beat Rhythm	Normal beats and rhythms	128	https://physionet.org/content/nsrdb/1.0.0/
BIDMC-Congestive Heart failure (CHF) database [51]	2 leads	15 two-channel 20-h recordings	Beat	CHF (NYHA class 3–4)	250	https://physionet.org/content/chfdb/1.0.0/
Normal sinus rhythm RR interval database [52]	2 leads	54 two-channel half-hour recordings	Beat	Normal beats	128	https://physionet.org/content/nsr2db/1.0.0/

^1^ All website URLs are accessed on 25 October 2022.

## 5. Interpretable Machine Learning (IML)

The need to determine the rationale behind the output decisions of the ML models began in the 1970s [60]. However, considerable advancements in the field of IML were attained in the last few years. Nevertheless, its conceptual foundation is still underdeveloped [61].

Currently, there is no well-established mathematical definition for the interpretability of ML models.It can also be called explainable artificial intelligence (XAI), and there is no well-agreed definition [62]. However, Murdoch et al. [63] defined the focus of an IML as “… the extraction of knowledge from an ML model concerning relationships either contained in data or learned by the model *…*”. According to their definition, knowledge is relevant if it provides insight for a particular audience in a given context. Based on the problems to be solved and users that use the output of an IML, this insight can be in the form of visual presentation, human-understandable languages, or mathematical equations.

### 5.1. Taxonomy of IML

**Figure 6:**
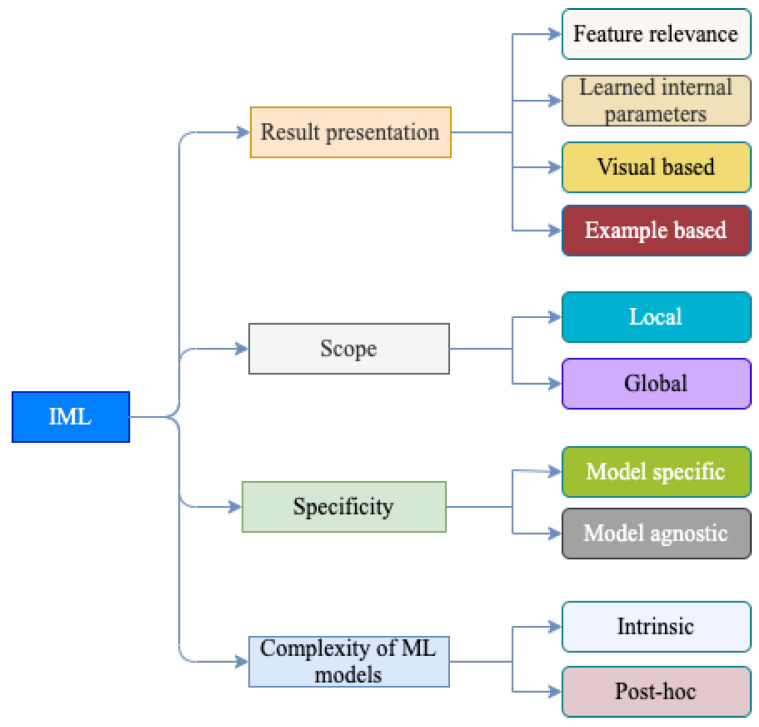
Taxonomy of machine learning interpretability.

When explaining the output and the behavior of ML models, different explanation techniques have been proposed in the literature. Based on discussions in the literature [39,40,62,64,65], in this article, we propose a taxonomy for IML techniques as shown in Figure 6. Here, the classification of IML techniques is based on their interpretation result presentation, scope, model specificity of the method, and the complexity of the ML model. However, the IML technique can hold a place in more than one of the classes in taxonomy. In subsequent sections, a detailed elaboration is provided based on the taxonomy given in Figure 6. In addition, the main concepts behind IML techniques and their usage for an ECG signal-based heart disease diagnosis of the heart are subsequently discussed.

### 5.2. Result Presentation in IML

In IML, there are various ways of presenting the results of the interpretation method that can provide insightful information to the user. Some result presentation methods include feature relevance, the model’s learned internal parameters, visual-based explanations, and example-based explanations.

#### 5.2.1. Interpretation Result Presentation Using Feature Relevance

Feature relevance-based ML model explanation is a technique used for interpreting the model’s output after the model training process. This technique provides a score on the contribution of each feature to the prediction output of the trained model [62,65]. Mathematically, it is possible to give the score for the feature contribution in the model output in terms of the input/output behaviors of the model. Thus, in the feature relevance-based explanation, the explanation is quantified using input features, $x:=(x_{1},\ldots ,x_{M})$ and the degree to which a given input feature $x_{i}$ contributes to the output of the model $f(x_{1},\ldots ,x_{M})$. Several techniques use future relevance to explain the AI models. However, this sub-section briefly discusses SHapley Additive exPlanations (SHAP), local interpretable model-agnostic explanations (LIME), and permutation feature importance.

##### SHapley Additive exPlanations (SHAP)

SHapley Additive exPlanations are derived from game theory; the SHapley values explain the marginal contribution of each player to the team. In interpreting ML models, these SHapley values indicate the contribution of each feature for a given black box model’s prediction or classification output. In determining the feature importance in the model output prediction or classification, SHapley values can be calculated depending on the complexity of the ML model. As a result, there are different techniques for determining SHaplely values, such as linear SHAP, kernel SHAP, and Deep SHAP [66,67]. The linear SHAP explains the feature importance in linear ML models. Given S⊆F, where *S* is a subset of all features $F=\{X_{1},X_{2},\ldots ,X_{k},\ldots ,X_{M}\}$, where $X_{k}$ represents features of a dataset at $k^{th}$ column in a dataset of size NxM. The contribution of feature $X_{i}$ to the output of a model *f* is performed in two different ways. First, the model training is underway with the presence of feature $X_{i}$, and the resulting model is represented as $f_{S\cup \{i\}}$, then it is retrained without the feature $X_{i}$, which is represented as $f_{S}$. Secondly, the originally trained model *f* helps to obtain both $f_{S\cup \{i\}}$ and $f_{S}$. Then, the SHapley value, $\phi_{i}$, for the feature $X_{i}$ is determined using Equation (1) [66]:

(1) $$\phi_{i}=\sum_{S\subseteq F\backslash \{i\}}\frac{|S|!(|F|-|S|-1)!}{|F|!}\left[f_{S\cup \{i\}}\left(x_{S\cup \{i\}}\right)-f_{S}\left(x_{S}\right)\right]$$

where $x_{S}$ represent the input feature values in a set *S*, $f_{S}\left(x_{S}\right)$ represents the marginal value of *f* for the features present in *S*, and $f_{S\cup \{i\}}\left(x_{S\cup \{i\}}\right)$ denotes the marginal value of *f* for the feature values present in *S* plus feature $X_{i}$. Thus, Equation (1) computes the disparity over all possible subsets S⊆F\{i} weighed by the number of features in the *S* from the total number of features, *F*.

Though the interpretation obtained from the SHapely values of the features can be comprehended and thoroughly tested for interpreting ECG-based ML models [68,69,70,71], the SHapley technique still has limitations. The major challenge is the computational burdens associated with calculating SHapley values for all feature subsets where the computational complexity is exponential [72]. In addition, it does not consider the correlation between the features. Instead, it takes all features as independent [66,73]. However, to mitigate these limitations, techniques, such as restricting the subset permutation using the causal relationship of features [74] and incorporating the constraint of correlations among feature values [75,76] have been proposed.

Moreover, to overcome the computational expensiveness of Equation (1), kernel SHAP [72], and treeSHAP [77] have been introduced. However, the computational complexities of SHAP-based post hoc model explanation techniques are still expensive. In addition, they can be tricked to rationalize decisions made by an unfair black box ML model; that is, they can be fooled [78].

##### Local Interpretable Model-Agnostic Explanations (LIME)

LIME is initially introduced by Ribeiro et al. [79], LIME approximates complex non-linear ML models with a locally interpretable surrogate model to explain which features hold the greatest contribution to the output of the black box ML model. This approximation relies on the assumption that complex models are linear on the local scale. Thus, approximating the complex model in the vicinity of individual instances to be explained may be feasible. This neighborhood significance is measured by the penalty function $\pi_{x}\left(z\right)$ that measures the proximity between perturbed instances, z∈R, around an instance feature vector, *x*. Thus, given *f*, a black box ML model to be explained, and *g* being a surrogate model best approximates *f* among a class of potential interpretable models *G*, i.e., g∈G. The explanation ξ(x) for an instance feature vector *x* produced by LIME is obtained by minimizing the objective function $L\left(f,g,\pi_{x}\right)+\Omega \left(g\right)$, as given in Equation (2) [79]:

(2) $$\xi \left(x\right)=\underset{g\in G}{\mathrm{argmax}}\;\left(L\left(f,g,\pi_{x}\right)+\Omega \left(g\right)\right)$$

where L is a locality-aware loss function for measuring how *g* is unfaithful in closely resembling *f* in the locality defined by $\pi_{x}$ and Ω(g), a measure of *g*’s complexity.

LIME uses a set of $d^{{}^{\prime }}$ interpretable representation features $x^{{}^{\prime }}\in {\left\{0,1\right\}}^{d^{{}^{\prime }}}$ that are sampled from the original feature space of the dataset, x∈X. By using binary vector represented perturbed instances $z^{{}^{\prime }}$ around non-zero elements of $x^{{}^{\prime }}$, a label for the explanation model, f(z), is obtained. The mapping of the binary vector representation of features to the original real-valued representation is performed via a mapping function $h_{x}$, such that $h_{x}:z^{{}^{\prime }}\to z$, i.e., $z=h_{x}\left(z^{{}^{\prime }}\right)$. Thus, using this dataset, *Z*, of perturbed samples with their labels, i.e., $\left\{\left(z^{{}^{\prime }},f\left(z\right)\right)\right\}$, the locality-aware loss function is defined as Equation (3) [79]:

(3) $$L\left(f,g,\pi_{x}\right)=\sum_{z,z^{{}^{\prime }}\in Z}\pi_{x}\left(z\right){\left(f\left(z\right)-g\left(z^{{}^{\prime }}\right)\right)}^{2}$$

Few pieces of literature have attempted to show the applicability of LIME in interpreting ECG signal-based heart disease classification ML model outputs [80,81]. LIME provides an easily understandable explanation, although it depends on the complexity of the local surrogate models. The interpretations made by the local surrogate models use features sampled from the original dataset. This process adds to the importance of LIME techniques, specifically when complex features are employed to train the black box ML model. However, the feature importance scores in a LIME do not add up to give the prediction probabilities that create ambiguity. Moreover, they do not deliver a global explanation of the learned complex ML model over the entire spectrum of feature values. In addition, the random perturbations of feature instances left the LIME techniques to suffer from the instabilities that pose challenges in reproducing the explanations. Furthermore, LIME can be manipulated to hide biases [78]. As a result, different techniques have been proposed in the literature to mitigate this instability and the resulting unfaithfulness of LIME [82,83,84,85].

##### Permutation Feature Importance (PFI)

PFI measures the change in the performance of the black box ML model while shuffling any given feature of the test dataset. Thus, PFI interprets the black box ML model by describing the contribution of a feature in the ML model’s output accuracy [86]. Given a trained model *f*, such that $f\left(x^{\left(i\right)}\right)\approx y^{\left(i\right)}$, where $x^{\left(i\right)}=\left(x{{}_{1}}^{\left(i\right)},x{{}_{2}}^{\left(i\right)},\ldots ,x{{}_{j}}^{\left(i\right)},\ldots ,x{{}_{M}}^{\left(i\right)}\right)$ are feature vectors and $y^{\left(i\right)}$ is a target of the $i^{th}$ instance. The PFI calculates the contribution of a given feature *j* in predicting $y^{\left(i\right)}$ as indicated in Equation (4) [87,88]:

(4) $$PFI\left(f,j\right)=\frac{1}{nk}\sum_{i=1}^{n}\sum_{l=1}^{k}\left[L\left[y^{\left(i\right)},f\left(x_{j}^{{\left(\tau_{l}\right)}^{\left(i\right)}}\right)\right]-L\left[y^{\left(i\right)},f\left(x^{\left(i\right)}\right)\right]\right]$$

where $\tau_{l}$ is a random permutation vector of instances in a dataset, $D={\left\{x^{\left(i\right)},y^{\left(i\right)}\right\}}_{i=1}^{n}$, with *n* instances for l=1,…,k permutations. L is a loss function linking the model output f(x) to the target pair *y*. Thus, $L[y^{\left(i\right)},f\left(x_{j}^{{\left(\tau_{l}\right)}^{\left(i\right)}}\right)]$ is the loss function linking the perturbed output of the model $f\left(x_{j}^{{\left(\tau_{l}\right)}^{\left(i\right)}}\right)=f\left(x{{}_{1}}^{\left(i\right)},x{{}_{2}}^{\left(i\right)},\ldots ,x{{}_{j}}^{{\left(\tau_{l}\right)}^{\left(i\right)}},\ldots ,x{{}_{M}}^{\left(i\right)}\right)$ to the target $y^{\left(i\right)}$ with respect to the perturbed feature $x_{j}$ and $L[y^{\left(i\right)},f\left(x^{\left(i\right)}\right)]$ gives a baseline loss linking the baseline output of the model and $f(x^{\left(i\right)})$ to the target pair $y^{\left(i\right)}$ for the instance *i*.

PFI has been experimented with to explain the classification output of ML mo; PFI can give model-agnostic global insight into the black box model, *f*. It also takes into account the dependency between features while determining their importance. In addition, it avoids retraining a model with a different subset of features, which saves time and even circumvents from reaching a new model due to the retraining process. Furthermore, the computational complexity associated with PFI is small enough to make the implementation easy. However, PFI needs a labeled ground truth of a given instance to calculate the feature importance. This limitation allows PFI to be used only during the model’s development, i.e., in the training and testing of an ML model. Likewise, in situations where strongly correlated features exist in a dataset, the result from PFI may be biased to the extent that less important features can take the highest importance value [89].

#### 5.2.2. Interpretation Result Presentation by Learned Internal Parameters of the Model

Explaining the internal learned parameters of the model is a commonly used interpretability technique in inherently transparent machine learning algorithms. For instance, in tree structures, the learned parameters include the features and splitting criteria [90]. This form of a result presentation is also used in deep learning models, such as interpretable filters of a CNN model [91].

Tree-based ML models, including decision tree, random forest, xGboost, and AdaBoost, techniques work by splitting the dataset using criteria, such as Gini impurity, mean squared error, and information gain, based on the feature value of the dataset. Each splitting creates different subsets from the dataset of the final, intermediate, and first subsets, respectively, called leaf nodes, split nodes, and root nodes [64,90,92]. Mathematically, the predicted instance, $\hat{y}$, obtained from the leaf node is represented in terms of feature *x*, as given in Equation (5) [92]:

(5) $$\hat{y}=\sum_{l=1}^{k}\mu_{m}I\left\{x\in R_{m}\right\}$$

where $\mu_{m}$ is the average value of all elements present in the subset $\left(R_{m}\right)$, $I\{x\in R_{m}\}$ is a binary identity function that gives 1 if *x* is in the $R_{m}$ subset, or else it returns 0. As stated earlier, the criteria used to generate the $R_{m}$ subsets can be the Gini impurity index, mean squared error, or information gain based on the problem and data type of the dataset.

In tree-based ML models, the learned parameters, including the splitting threshold values of a feature, the Gini impurity index value, and the number of data points of the model are explained more easily. However, as the tree depth increases, the interpretation becomes difficult, and the model becomes opaque. In addition, the interpretation of truthfulness is affected by the poor generalization properties of the tree models themselves, where most tree-based ensemble models lack stability, especially while modeling complex interactions among several features [64,93,94,95,96].

#### 5.2.3. Interpretation Result Presentation through Visual Explanation

One way of interpreting the prediction output of the black box machine learning model is by highlighting the important segments in the data that contribute the most to the decision of an ML model [97]. Visual explanation-based result presentation techniques have been extensively tested in interpreting black box machine learning classifiers in an ECG signal-based heart disease diagnosis. Some of them include class activation map-based techniques [98,99,100,101], saliency maps [102,103], layer-wise relevance propagation [104], occlusion maps [102], and attention maps [105,106,107,108]. Moreover, LIME [80], and SHAP [70,109,110,111] are used to explain the decision of the ML techniques by visually representing the important regions of an ECG signal, which contributes most to the decision. To acquaint the reader with the pros and limitations of these techniques, a brief discussion on some of the methods is presented as follows.

##### Class Activation Maps

The class activation map technique introduced by Zhou et al. [112] provides a visual explanation by localizing the important regions in input data that play major roles in the decisions of ML models. In class activation, the descriptive regions of input data that an ML model used for classification are highlighted [113]. The class activation map calculates the contribution of units ($L_{ij}^{c}$) in the last layer activation filter map ($F_{ij}^{k}$) of the convolutional layer for the class prediction score ($y^{c}$) of the output layer. The CAM technique proposed by Zhou et al. [112] used global average pooling (GAP) and fully connected layers (FC) to obtain $L_{ij}^{c}$. In [112], $F_{ij}^{k}$ and $y^{c}$ have a linear relationship as given in Equation (6).

(6) $$y^{c}=\sum_{k}w_{k}^{c}\sum_{i}\sum_{j}F_{ij}^{k}$$

where $w_{k}^{c}$ is the weight of the FC for filter *k*; classes *c*, *i*, and *j* are indices of the last feature map units; *c* is the class category; and *k* is a filter index.

The main aim of CAM is to find the contribution of the last feature maps that satisfy $y^{c}=\sum_{i,j}L_{ij}^{c}$. Thus, the contribution of each unit in the last feature map, $L_{ij}^{c}$, can be obtained from Equation (6), as shown in Equation (7):

(7) $$L_{ij}^{c}=\sum_{k}w_{k}^{c}F_{ij}^{k}$$

In a single-dimensional time series signal, such as an ECG signal, the class activation map for class *c* at the specific temporal instance *t* is as indicated in Equation (8):

(8) $$L_{t}^{c}=\sum_{k}w_{k}^{c}F_{t}^{k}$$

where $F_{t}^{k}$ is the activation of filter *k* in the last conventional layer at the temporal instance *t*, and $L_{t}^{c}$ indicates the importance of the activation at the temporal location *t* leading to the categorization of a signal into class *c*.

CAM has been used for interpreting an ECG signal classification result of a convolutional neural network [114]. Accordingly, it allows the visualization of segments of an ECG signal that the classification model mainly uses in its decision. Techniques, such as Grad-CAM [98,99,115,116,117,118,119,120,121,122,123], Grad-CAM++ [101,124], and guided Grad-CAM [125] have been proposed in the ECG signal-based heart disease classification. However, the linear layers vanish the non-linearity of deep classifiers. In addition, the integration of CAM changes the network architecture and needs retraining [126]. Moreover, these gradient-based CAMs suffer from a gradient saturation problem that results in inaccurate localization of relevant regions. In addition, the localization of the descriptive signal part is highly affected by small perturbations of the input signal. Furthermore, the explanation is noisy and contains discontinuities [126].

##### Saliency Maps

Feature saliency map highlights the regions of a signal that are most relevant for categorizing the input signal into a given class. The saliency map can be built using gradients of the output, $y_{c}\left(x\right)$, of an ML model over the input, x, for the class *c* [102]. The idea is that the class score $y_{c}$ can be approximated by using the first-order Taylor expansion as given in Equation (9):

(9) $$y_{c}\left(x\right)\approx w^{T}x+b$$

where *b* is a scalar, and w, as indicated in Equation (10)), is the gradient that provides an explanation for the model classification outcome:

(10) $$w=\frac{\partial y_{c}\left(x\right)}{\partial x}$$

Among other techniques, the saliency map can be generated using guided backpropagation where the gradient of each neuron is calculated and those with the highest gradient values are activated to form a heatmap [103]. The heatmap shows the most salient parts of the signal that contribute most to classifying the input x to class *c*.

Saliency maps were experimented with for explaining complex ML models in ECG signal-based heart disease diagnosis [102,103,127,128]. Although the backpropagation gradient saliency map can visually enhance regions of the input signal that contribute the most to classification, it has certain limitations. At first, the backpropagation saliency suffers from a gradient saturation problem mainly because saliency maps are based on input sensitivity [129]. Next, the generated gradient heatmap often does not explain the direct relation to the classifier’s decision. Instead, it only indicates the important signal segments used by the model for classification [130]. More importantly, the saliency method is susceptible to small shifts in the input signal so that its explanation may not be reliable [131].

##### Layer-Wise Relevance Propagation (LRP)

An LRP provides an explanation through the decomposition via computing a relevance score ($R_{n}$) based on the contribution of each input element $x_{n}$ for the model’s (*f*) output prediction y=f(x), given the input sample, $x=[x_{1},\ldots ,x_{n},\ldots ,x_{N}]$. Thus, an LRP explains the ML model’s output by attributing relevant values to the essential components of the input by tracing back the trained model layer by layer, starting from the final output node [132]. This layer-by-layer relevance propagation holds the layer-wise conservation property, given that *i* and *j* are neurons at two consecutive layers of a neural network, *l* and l+1, respectively. The overall sum of the *i*th neuron’s relevance score sums to $R_{i}^{\left(l\right)}$, such that relevance conservation property is maintained:

(11) $$R_{i}^{\left(l\right)}=\sum_{j}R_{i\leftarrow j}^{\left(l,l+1\right)}\;\mathrm{such}\;\mathrm{that}\;i\;\mathrm{contributes}\;\mathrm{to}\;j$$

The propagation of relevant scores $R_{j}$ of layer l+1 onto neurons of the *l* layer can be achieved using different types of rules. Moreover, different rules can be used at each layer of the network architecture [133]. One of the simplest rules is given in Equation (12) [132]:

(12) $$R_{i}=\sum_{j}\frac{a_{i}w_{ij}}{\sum_{0,i}a_{i}w_{ij}}R_{j}$$

where $a_{i}$ is an activation of the neuron *i*, $w_{ij}$ is the weight connecting neuron *i* to neuron *j*, and $\sum_{0,i}$ indicates the sum over all neurons *j* in the *l* layer. Moreover, the rule satisfies the basic properties in which deactivated neurons, neurons with no connection, and zero weight has no relevant value.

LRP has been used for interpreting the DL model output through heat mapping the relevant regions of the input that contribute most to the output prediction. Having fewer noises around the target class and the capacity to show the part of a signal that negatively contributes to the output, LRP is superior over gradient-based explanation techniques [133,134]. However, the heatmap produced by an LRP is still noisy due to the initialization of the non-target class to zero relevance value. Moreover, it has a limitation in discriminating targets that produce identical heatmaps for different entities in an input signal [135]. Furthermore, the selection of propagation rules is problem-dependent, and obtaining the best parameters is trivial [136]. As a result, different techniques, such as contrastive LRP [137], selective LRP [135], and a softmax–gradient LRP [138] are being proposed in the literature to alleviate these challenges.

##### Occlusion Map

The occlusion map is one of the attribution-based techniques where the model output is explained by changing part of the input data with different values [139]. The input can be altered on a specific location, for instance, in a time series signal such as an ECG with total *h* time points, the alteration can cover certain time step durations (d) with an occlusion window of (w). For a signal $x=\{t_{1},t_{2},\ldots ,t_{h}\}$, the locally altered signal $\left(\hat{x}\right)$ can be obtained as follows Equation (13) [139]:

(13) $$\hat{x}=\left(x⊙m_{1}\right)+o_{v}m_{2}$$

where $m_{1}$ and $m_{2}$ are masks that complement each other, i.e., $m_{2}=\neg m_{1}$ and $o_{v}$ are the occluding values. The values for $m_{1}$, $m_{2}$, and $o_{v}$ are determined based on the required modifications on *x*.

The occlusion-based ML model’s interpretation algorithms are simple to implement. Moreover, it can measure the marginal effects of each windowed region of the input signal given that the segments of the input are independent [140,141]. In addition, the occlusion method is used to interpret the output of non-differentiable ML models, unlike gradient-based explanation techniques [102]. However, similar to other perturbation-based model output explanation methods, such as LIME and SHapley value maps, the computational complexity associated with the input occlusion is high [142,143].

##### Attention Mechanisms

Attention mechanisms are commonly used in time-series data because of their ability to improve the limitation of traditional encoder–decoder-based models [106,144]. The attention mechanism can be incorporated into ML networks and it allows the ML model to focus on specific regions of an input signal that contributes most to the output prediction [105,106,144,145,146,147,148]. Moreover, domain-specific knowledge can be integrated to guide attention mechanisms so that the contribution of each segment of a signal in the model’s classification output is captured [145].

The attention mechanism takes the encoder output (latent vector) as the input and performs three consecutive computations, which are alignment scoring ($e_{ij}$), computing attention weights, and attention score vector computation, as given in Equation (14), Equation (15), and Equation (16) [149], respectively.

(14) $$e_{ij}=a\left(s_{i-1},h_{j}\right)$$

where *a* is an alignment model whose score $e_{ij}$ measures how well the input around position *j* of the encoder’s hidden state $h_{j}$ matches the previous decoder hidden state $s_{i-1}$ at position *i* just before emitting. Then, the attention weight score ($\alpha_{ij}$) of each $h_{j}$ is computed by applying an activation function, for instance, the softmax activation function, on the alignment score as shown in Equation (15).

(15) $$\alpha_{ij}=\frac{exp\left(e_{ij}\right)}{\sum_{k=1}^{T}exp(e_{ij})}$$

where *T* is the number of the encoder’s hidden states. Finally, the attention score vector, which is the output of the attention mechanism, is computed as a weighted sum of all encoder hidden states, as shown in Equation (16).

(16) $$c_{i}=\sum_{j=1}^{T}\alpha_{ij}h_{j}$$

Based on the techniques employed for generating attention scores, attention mechanisms are broadly classified into deterministic attention and stochastic attention [150]. In the case of a deterministic, attention scores are calculated as the weighted sum of all hidden states, whereas, in stochastic attention, attention scores are determined by selecting one of the hidden states, $h_{j}$.

The attention mechanism introduces the model’s output interpretability scheme, in addition to improving the performance of the ML model’s ECG signal-based heart disease classification [105,106,107,108,144]. However, the computational complexity associated with an attention mechanism is one of the limitations that need to be improved [144].

#### 5.2.4. Interpretation Result Presentation Using an Example-Based Explanation

Example-based ML model’s output explanation techniques inform end-users about the ML model’s output prediction on a particular sample instance by selecting example data from the training set [62,151]. The concept in an example-based explanation technique is that if two data instances ($X_{i}$ and $X_{j}$) are similar and the ML model’s (*f*) output for input data instance $X_{i}$ is $y=f(X_{i})$, then the model output for a data instance $X_{j}$ is also *y*.

Example-based ML output explanations include counterfactual [152,153] and adversarial examples [154]. Moreover, inherently interpretable (transparent) shallow ML algorithms include the k-nearest neighbor (KNN) [65,155] work based on an example-based approach. These techniques work through minimizing a loss function, commonly a distance metric between the instance to be explained z and its perturbed form $z^{\prime }$. In this method, the ML model’s output is explained by finding the extent of perturbations on the input instance that brings changes to the outcome of the ML model. Formally, given an ML model f:Z→Y, a data instance z∈Z with model output y=f(z), and the desired model output target $y^{\prime }\in Y\backslash \left\{y\right\}$, a counterfactual explanation solves the objective function, *d*, given in Equation (17) [152]:

(17) $$\underset{z^{\prime }\in Z}{\mathrm{minimize}}\;d\left(z^{\prime },z\right)\;s.t.\;f\left(z^{\prime }\right)=y^{\prime }$$

where *d* is any distance metric.

Example-based explanation techniques highlight part of an input instance or feature values changed to give the target class $y^{\prime }$. In other words, the explanation gives the difference between z and $z^{\prime }$, such that $f\left(z\right)\neq f\left(z^{\prime }\right)$. In addition, an example-based explanation is easily implemented because of the objective function that can be easily optimized [156,157]. However, there will be more than one example for a single sample instance that results in a lack of obtaining a unique explanation for a particular input instance. Moreover, several challenges need to be addressed, including limitations in visualizing results [157].

### 5.3. Scope of IML Techniques

Based on whether the explanation is for a specific sample instance of the input or via comprehending how the complete model works, IML models are classified as locally or globally interpretable. Local interpretable methods are scoped to explain how the individual output of an ML model is done on a single instance input. On the other hand, globally scoped interpretable methods explain the whole logic of the model and the entire reasoning follows for all possible outcomes of the model [39,62,63].

Local model interpretation methods focus on answering ’why an ML model makes a given specific prediction?’. Moreover, these methods can reveal the effects of a specific segment of input instances or feature values on the output of the model [62,84]. Thus, these techniques help to understand the causal relations between specific input instances and their corresponding ML model outputs [39]. However, the explanation obtained from these techniques is valid only for a single input instance and does not generalize. In addition, the explanation result obtained from these techniques lacks stability. That means the explanation generated through consecutively running these techniques may result in a different outcome. Furthermore, the local surrogate model may spuriously approximate the complex ML models, i.e., the explanation outcome may have no real connection with the ML model [158,159].

On the other hand, global model interpretation methods focus on answering ’how an ML model makes a prediction?’. These methods can try to understand how subsets of the model influence the model’s decisions. Global interpretability can be achieved through training interpretable constraints together with the input data [39]. In addition, it can also be achieved by demonstrating the statistical contribution of each feature in the decision of the underlying black box model. Furthermore, the global explanation can also be obtained by capturing representation at the intermediate layers of complex DL models. Thus, these techniques help to understand the inner working mechanisms of ML models and increase the model’s transparency [39]. However, globally scoped interpretation techniques often miss explaining a model output for specific input instances. However, different methods have been proposed in the literature for obtaining a global explanation of the black box model through aggregating local explanations [160].

### 5.4. Specificity of IML Techniques

Based on their capacity to transcend for different ML models, interpretability techniques categorized into model-specific and model-agnostic [62] techniques. The model-specific interpretation techniques are used to explain specific model classes and the use of internal model parameters to explain the ML model’s output [39]. On the other hand, model-agnostic IML techniques provide explanations independent of internal model parameters. Instead, they give explanations by relating the input of a black box ML model to its output [65].

Model-specific explanation techniques not only explain the model outputs based on the model characteristics but also help in improving the efficiency of the ML model by investigating the characteristics. Moreover, model-specific interpretation techniques have high translucency in which they can rely on more information to generate an explanation [62]. However, they are limited to a specific model and are less portable to explain other models. On the other hand, model-agnostic interpretable techniques are independent of the model to be explained and can be applied to any model [65]. However, due to the approximation and assumptions made in constructing model-agnostic interpretation techniques, their explanation results may become less accurate and even vulnerable to adversarial attacks [65,78,154]. In addition, it may be difficult to faithfully detail the explanation produced by model-agnostic methods, as to how they truly reflect the decision-making processes of the ML model [39]. Furthermore, the computational complexities of model-agnostic techniques, such as SHapley values, grow exponentially as the number of input features increases [159].

### 5.5. Complexity of ML Models

Based on the complexity of an ML model to be explained, the interpretability methods are categorized into intrinsic and post hoc. In intrinsic interpretability, the explanation is based on understanding how the ML model works. On the other hand, in post hoc interpretability, the explanation is provided by extracting a piece of information from a trained complex black box ML model [62].

The intrinsic explanation methods used for ML models have simple architecture by design and provide self-explanatory results. However, these ML models cannot be used to solve complex problems and suffer a lot from capturing nonlinearity in the data. In the literature, methods have been proposed to mitigate the trade-off in reducing the model performance for interpretability. One of the methods is adding semantically meaningful constraints to complex models to improve interpretability without a significant loss in the performance [91]. Moreover, domain-specific knowledge can be integrated with complex ML models through attention mechanisms to improve interpretability, as discussed in Section 5.2.3 of this article.

The post hoc explanation methods are usually applied after the ML model is trained and provide an explanation without modifying the trained model. Moreover, the complex ML model can be approximated by surrogate models, such as decision trees and shallow neural networks. These surrogate models provide a global post hoc model-agnostic explanation by mimicking the complex ML model [161,162,163]. These techniques are much more flexible and can switch to explain different black box ML models. However, the post hoc methods compromise the fidelity of the explanation. In addition, they may fail to represent the behavior of the complex ML model [39].

### 5.6. Summary of Taxonomy of IML Techniques

Both globally and locally scoped interpretable techniques can be ML model specific or model agnostic and used for intrinsic model explanations or post hoc explanations [39]. IML techniques that are commonly used in ECG-based heart disease diagnoses are given in Table 6.

## 6. Performance Evaluation of Interpretability Methods

The black box nature of ML models has been a challenge in implementing ML-based solutions in healthcare and other critical tasks where knowing the reason behind the ML decision is essential. As a result, several ML model interpretability techniques have been proposed in the literature, as discussed in Figure 5 of this paper, to mitigate these challenges and improve the ML model’s output explanation. Moreover, the performance of IML techniques in explaining the complex ML model should be measurable so that users can easily pick the best technique for a particular problem. In addition, researchers can compare and improve the limitations of IML techniques. Carvalho et al. [40] and Zhou et al. [166] provided a detailed discussion on IML technique performance evaluation methods and metrics. They indicated the difficulties in finding a fit for all evaluation metrics for measuring the performances across all IML techniques and domain problems. Thus, this section focuses on the methods and metrics used in the literature for measuring the explanation of the IML techniques in an ECG signal-based heart disease diagnosis. We can broadly classify these metrics into qualitative and quantitative.

In qualitative explanation metrics, a human user (expert) evaluates the goodness of the explanation obtained from the IML method mainly through observation and compares it with clinical findings. However, most researchers claim their proposed technique sufficiently explains the prediction output of the black box ML model without validating their methods by human experts in the field. The quantitative metrics evaluate the expressiveness of the explanation result using metrics, such as attention score, Jaccard index, and performance decrease. However, it is worth noting that there are commonly agreed quantitative evaluation metrics for IML techniques [167].

### 6.1. Visual Observation

In a visual observation evaluation, the ML models are usually explained by showing segments of an ECG signal that contribute most to the ML model’s output prediction. This metric demands a human expert to visually inspect the explanation generated by the IML. Moreover, the metric can serve as a gold standard since the visual justification produced by IML techniques is easy to understand for physicians. However, validating an explanation using visual checking is time-consuming and does not guarantee complete insight into the underlying disease condition [147]. Table 7, Table 8 and Table 9 list the IML techniques evaluated using visual evidence. These visual explanations can be taken as a proof concept in which highlighting segments of an ECG can contribute to explaining a complex ML model output. However, these techniques cannot provide a reason for the question, ’why are these regions highlighted?’; this poses difficulties for physicians in understanding the explanation. In addition, IML technique evaluations through visual observation must incorporate human expert intervention for validating the explanation output. However, this is a highly challenging task due to the expense of preparing ground truth benchmarks for evaluation and the time requirement. As a result, except for Bleijendaal et al. [141], all articles reviewed in Table 7, Table 8 and Table 9 are not validated by human experts or cardiologists.

### 6.2. Feature Effect

This technique sometimes overlaps with the visual observational-based evaluation of explanations obtained from IML methods. For instance, some of the SHAP based techniques [70,80,110] discussed in Table 10 provide explanations for the model output by highlighting the segments of an ECG signal. However, these techniques focus on the contribution and association of ECG signal features for the ML model’s output prediction. Interpretations obtained from feature attribution-based IML techniques are often evaluated using the feature effect techniques by comparing the explanation results with prior domain knowledge. Thus, examining the feature effects requires human expert intervention to determine the explanation’s clarity and soundness [166].

### 6.3. Attention Score

The attention score evaluates the explanation performance of an IML technique quantitatively. Elul et al. [147] attempted to compare the performances of the attention mechanism and Grad-CAM IML techniques in explaining the ML model’s prediction output. In addition, they demonstrated that attention score assists in identifying the influential ECG tracing leads that have meaningful clinical information in diagnosing heart disease, such as AF, ST, and VT.

### 6.4. Jaccard Index

The Jaccard index, also known as intersection over union, is one of the most commonly used similarity measures that enable us to find the similarity among two finite sets *P* and *Q*. The Jaccard index has been used to measure the performances of computer vision models applied in various application domains [173,174,175,176].

As given in Equation (18), Neves et al. [80] measured the performance of their proposed IML method’s explanation results, showing the most relevant segments of an ECG signal $\left(W_{w}\right)$ against shapelet-based classifiers. Equation (18) computes the intersection divided by the union of the number of elements between two sets, shapelets and $W_{w}$ [80]. The value of Equation (18) is in the range of 0 and 1. J=0 indicates that there is no match between the shapelets and $W_{w}$, and J=1 indicates that shapelets and $W_{w}$ fully match.

(18) $$J\left(shapelets,W_{w}\right)=\frac{\left(shapelets\cap W_{w}\right)}{\left(shapelets\cup W_{w}\right)}$$

Neves et al. [80] uses the shapelet classifier [177,178] output as a ground truth to measure the performances of IML methods. However, it is worth knowing that the shapelet classifier has associated performance issues. Thus, the result obtained from Equation (18) may not faithfully measure the performance of the IML methods in reality.

### 6.5. Performance Decrease

In the performance decrease approach, first, the most relevant regions of an ECG tracing identified by the IML method $\left(W_{w}\right)$ are replaced from the original signal. Then, the performance of the black box ML model is recalculated [80]. To replace the relevant parts of the original ECG signal, techniques such as random perturbation, making the region zero, or swapping can be used [80].

The performance decrease-based approach does not need ground truth to measure the performances of IML techniques. Thus, IML method performance results obtained from this approach may not be feasible to be used in reality.

## 7. Discussion

**Figure 7:**
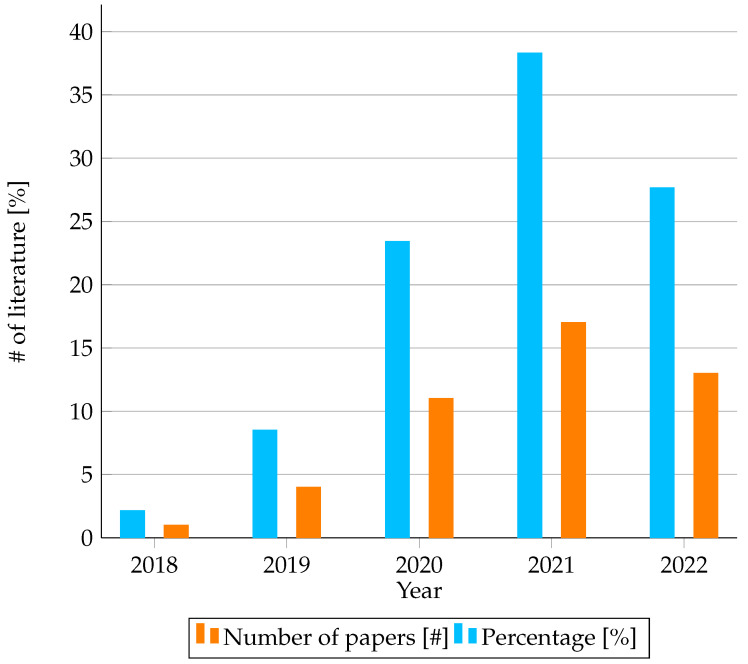
Yearly distribution of the reviewed research papers.

The non-invasive diagnosis test nature of an ECG and its associated lower cost has made it one of the most commonly used tools in heart disease diagnosis. However, most physicians, irrespective of their experience and specialty level, face challenges in accurately reading ECG tracings. This challenge often arises due to several types of heart disease, the indistinguishable manifestation of heart disease in an ECG tracing, and the variation of ECG tracings because of the patient’s age, race, and physical condition. Recently, ML-based heart disease classification techniques using ECG tracings have been proposed in the literature to aid physicians in reading an ECG tracing. However, the black box nature of ML techniques has left physicians from knowing the reason behind the ML model’s classification output and faithfully using the model’s results. As a result, different IML techniques have been suggested for explaining ML model outputs. As shown in Figure 7, the number of literary studies that proposed IML methods for interpreting the reason behind the result of the ML model’s heart disease classification (from an ECG signal) is increasing; this is an active research area.

**Figure 8:**
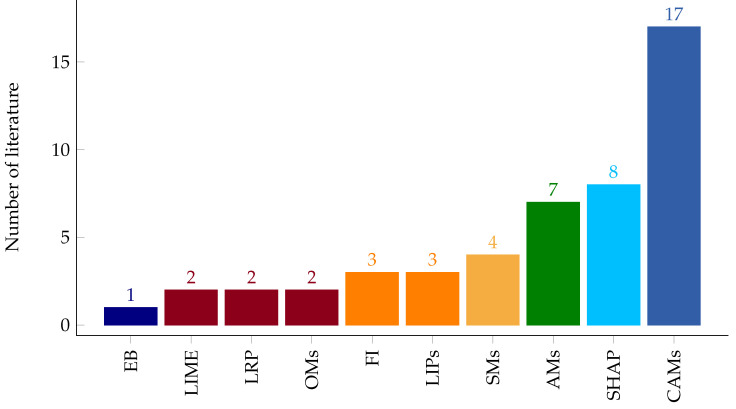
Type and number of reviewed IML methods.

This systematic review work presented a thorough investigation of IML methods used in explaining outputs of heart disease classification results of black box ML models. Among the IML techniques proposed in the literature, the class activation maps and their variants, such as Grad-CAM, guided Grad-CAM, and Grad-CAM++ took the lion’s share, as shown in Figure 8. These techniques localize in the form of heatmaps, i.e., the regions of an ECG signal where the black box ML model is used in its classification output. However, apart from localization inaccuracy, the explanation presentation technique via the heatmap might not be well understood by expert physicians.

Similarly, most of the IML techniques proposed in the literature for explaining black box heart disease classification ML models attempted to localize segments of an ECG signal that the ML used for output prediction. However, for a physician who has no exposure to the concepts of IML or machine learning, these types of explanations may not help in obtaining an evidence-based diagnosis. In addition, the performances of these IML techniques were not measured against ground truth, partially because of the unavailability of the annotated dataset and commonly agreed-on quantitative metrics. For instance, the ECG heart disease dataset presented in Table 4 was annotated only by disease types and did not incorporate clinical reasons or findings. As per our knowledge, no publicly available ECG heart disease dataset contains the clinical descriptions for categorizing the ECG tracings into their respective disease class. Moreover, most IML methods proposed in the literature for explaining the ECG signal-based heart disease ML classification outputs are adopted from computer vision and other applications where the model training data are either images or tabular formats.

**Figure 9:**
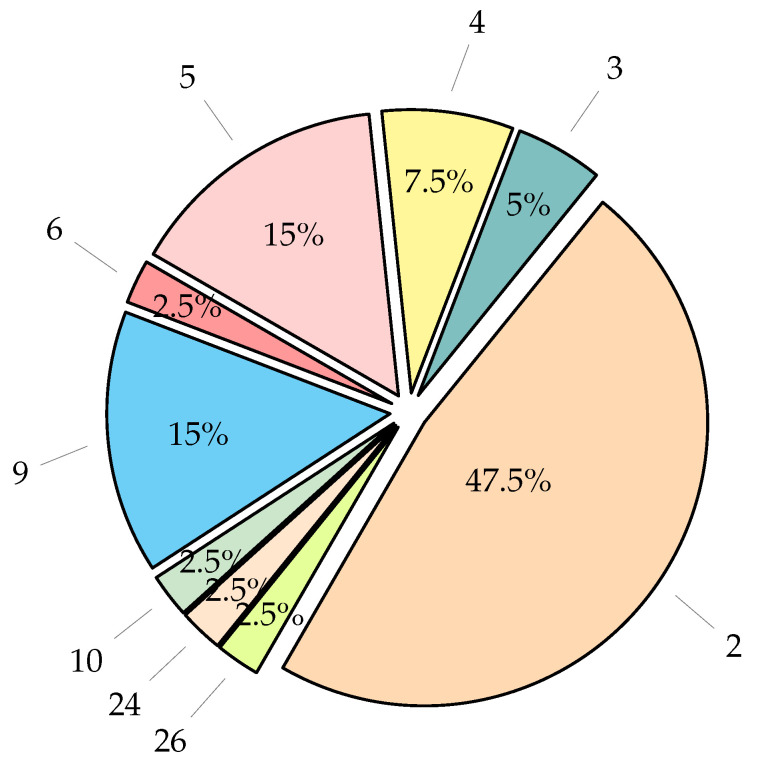
Distribution of reviewed IML methods with respect to the number of disease classes.

Integrating IML methods in the workflow of the ML model development for heart disease classification from an ECG signal is in its infancy stage and not well tested. As shown in Figure 9, almost half of the published articles attempted to integrate and test their proposed IML methods to explain the classification outputs of only two disease conditions.

## 8. Challenges and Future Direction

The benefits of developing a ML model that classifies heart disease from an ECG signal are immense. However, the black box nature of these models coupled with ECG signal complexities pose difficulties with their integration into clinical diagnosis workflows. As a result, IML techniques are proposed in the literature to explain the classification outputs of the black box ML models. However, to reap the application of IML in interpreting the ECG signal-based ML models, existing challenges should be addressed. These challenges include limited concepts in choosing and designing IML methods, a lack of well-defined use cases, and the absence of standardized performance evaluation metrics.

First, the process of choosing a method that suits a particular application (from existing IML methods) is a challenging task. In addition, designing new techniques will require the collaboration of interdisciplinary experts from different domains. This is partially because the output of the IML methods should be usable by human experts to improve their faith in the ML classification model’s results.

Secondly, the use cases of IML methods in interpreting the classification output of an ECG signal should address the physician’s needs. The existing IML techniques attempted to merely highlight or give feature characteristics of the ECG segments. These techniques may not be well-understood by physicians. Thus, integrating physicians in the process of the IML method development aids in developing a use case where the explanation output of IML aligns with physician reasoning in diagnosing heart disease from ECG tracings.

Apart from the above two situations, the lack of commonly agreed-upon metrics used to measure the performances of IML methods poses a challenge in evaluating the quality of the proposed techniques. Thus, it is critical to strengthen the few existing practices and devise new metrics for measuring the performances of IML methods through rigorous testing.

## 9. Conclusions

Heart disease diagnosis from ECG tracings is difficult for physicians across different levels. This difficulty necessitates the intervention of ML models. However, the black box nature of these ML models and their limited performances have reduced their trustworthiness. Thus, the usefulness of interpreting the output of black box ML models is undeniable in earning the trust of physicians. Thus, in this systematic review work, we first identified the available heart electrocardiogram diagnosis datasets. Then, we discussed the taxonomy of IML methods in terms of the result presentation method, scope, specificity, and complexity of the ML model. In addition, we briefly examined these methods with their strengths and weaknesses. Furthermore, we present the progress made in integrating the IML methods in an ECG signal-based heart disease diagnosis through a few established performance evaluation metrics. Finally, we discussed the existing challenges in IML techniques and their mitigation options.

The main findings of this review work, in terms of the research questions listed in Section 3.1, are summarized as follows:

- *RQ1: Are there any freely available heart ECG signal datasets? What are their characteristics?*
  As discussed in Section 4, there are several annotated heart disease ECG tracing datasets in repositories. These datasets are composed of single-lead and 12-lead ECG tracings (sampled at different sampling frequencies). In addition, the number of recordings in the dataset and classes annotating heart disease also vary. Moreover, the disease classes in these datasets are not balanced. Furthermore, some annotations are at the heartbeat level and others involve whole ECG tracing. Above all, these repositories are not fit for developing and testing IML methods as they do not have clinical reasoning, such as location and morphological manifestations of abnormalities in ECG tracing.
- *RQ2: What are IML techniques and commonly investigated interpretable techniques in ECG signal-based heart disease diagnoses?*
  As discussed in Section 5, we identified IML methods and categorized them in a taxonomy to discuss their working principles and spot their gaps. These IML methods attempt to localize the regions of an ECG signal that contributes the most to the classification process. However, they have limitations, such as computational complexity, gradient saturation problem, lack of generalization, and susceptibility to input ECG signal perturbation.
- *RQ3: What is the overall progress and performance of IML algorithms in providing evidence-based heart disease diagnoses?*
  The proposed methods in the literature explain the ML model’s output in terms of visual presentation, feature importance, internal ML model parameters, and factual examples. However, the explanations provided are not easily understandable. In addition, due to the lack of commonly agreed-upon performance evaluation metrics and ground truth, the methods are not rigorously evaluated.
- *RQ4: Are there any limitations and challenges in IML-based heart disease classifications?*
  Section 8 clearly identifies the existing challenges, such as the absence of standardized evaluation metrics, lack of well-defined use cases, explanation clarity, and ground truth dataset. In addition, future directions are highlighted.

In conclusion, the promising results achieved so far should be strengthened by defining the use cases of IML methods together with expert physicians. In addition, new techniques should be designed, and existing ones need to be customized to achieve physician-level reasoning behind ML model decisions. Furthermore, the research community has to devise performance evaluation metrics to evaluate the IML methods. 

## Figures and Tables

**Table 1:** Summary of related works.

Article, Year of Publication	Contribution	Limitation
Abdullah et al. [32], 2021	Presented a comprehensive survey on the uses of IML techniques in healthcare; The paper presented an in-depth theoretical discussion of the existing well-known IML techniques.	Only a single piece of literature was reviewed that focuses on the application of IML on ECG signal-based heart disease classification; Limited discussion on how to evaluate the performance of IML techniques.
Xiong et al. [34], 2022	Reviewed the most popular deep learning algorithms for detecting and locating myocardial infractions.	Did not include a discussion on the interpretability of ML models used for myocardial infraction detection.
Somani et al. [35], 2021	Reviewed deep learning-based literature aimed at detecting and classifying five (5) types of heart disease from an ECG signal	Presented limited and shallow discussions on the interpretable model.
Rasheed et al. [36], 2021	Provided a comprehensive review of IML techniques	Reviewed single literature on IML-based ECG signal interpretation.
Yang et al. [37], 2022	Described the progress made in applying explainable AI in healthcare; Showcased the importance of explainable AI in clinical scenarios.	The review did not include literature on interpreting ML models designed for ECG signal-based heart disease classification.
Stiglic et al. [38], 2020	Discussed the applicability and importance of interpretability for healthcare applications	Gave more emphasis to feature importance-based explanations and few discussions were provided for other ML model explanation techniques Limited discussion on the pros and limitations of interpretation techniques.
Du et al. [39], 2019	Presented a clear overview of some of the existing IML techniques; Discussed challenges in the implementation and evaluation of IML techniques;	The review did not include literature on interpreting ML models designed for ECG signal-based heart disease classification.
Carvalho et al. [40], 2019	Explained how to evaluate the explanation quality of IML techniques; Outlined challenges to be addressed in the field of interpretable AI.	Focused on the societal impact of interpretable AI; Limited discussions on the IML techniques used in the healthcare field in general, and in ECG-based heart disease classification in particular.
Jin et al. [41], 2022	Provided a discussion on the pros and limitations of various IML techniques for general domain applications and of their adoption for healthcare; Discussed how to assess the credibility and trustworthiness of IML techniques.	The review did not include literature on interpreting ML models designed for ECG signal-based heart disease classification.

**Table 2:** Review questions with main motivations.

No.	Review Question	Aim to Answer
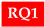	Are there any freely available heart ECG signal datasets? What are their characteristics?	Identify heart ECG signal datasets The characteristics, nature, and important features of ECG
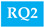	What are IML techniques and commonly investigated interpretable techniques in ECG signal-based heart disease diagnosis?	Identify and thoroughly discuss interpretable machine learning that is often used in classifying heart disease from an ECG signal
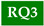	What is the overall progress and performance of IML algorithms in providing evidence-based heart disease diagnosis?	Identify the progress that has been made so far in providing evidence-based ECG signal interpretation using IML.
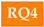	Are there any limitations and challenges in IML-based heart disease classification?	Identify limitations, challenges, and future directions in using an IML for evidence-based ECG signal interpretation

**Table 3:** Literature inclusion and exclusion criteria.

Inclusion Criteria (I)	Exclusion Criteria (E)
I1: Published between 2018 and 2022	E1: White papers, MSc. thesis, Ph.D. dissertation, magazines, and written other than English language
I2: The journal article should focus on one or more IML techniques in heart disease ECG signal interpretation	E2: Articles that focus on non-ECG heart diseases classification
I3: The study should clearly discuss the IML method	E3: The study is not focused on the interpretability or explainability of machine learning models
I4: The study should quantify the interpretability performance of the IML method	E4: Results and findings of the study are not clearly explained and plausible

**Table 6:** Summary of commonly used techniques for ML interpretation in ECG-based heart disease classification.

Technique	Scope	Specificity	Complexity	Result Presentation
LIME [80,81]	Local	Model-agnostic	Post hoc	Relevant features of an ECG are identified or highlighted regions of an ECG signal containing the relevant features.
Feature importance (FI) [80,164,165]	Global	Model-agnostic	Post hoc	Features that have meaningful clinical significance are identified based on their importance in the ML model’s output classification.
SHAP [68,69,70,71,80,109,110,111]	Local/Global	Model-agnostic	Post hoc	Rank the global feature importance of an ECG signal and provide a local explanation for the model classification output. Moreover, it can highlight descriptive morphological segments of an ECG signal.
Attention mechanisms (AMs) [105,106,144,145,146,147,148]	Local	Model-specific	Intrinsic	Visual explanation: uses attention weights to interpret classification or detection output by visually specifying the segments of the input signal.
Layer-wise relevance propagations (LRPs) [104,132]	Local	Model-agnostic	Post hoc	Highlights regions of the input signal to indicate the contribution of each region through the back-propagating relevance score from the ML model’s final output.
Occlusion Maps (OMs) [102,141]	Local	Model-agnostic	Post hoc	Identify segments of an ECG signal by replacing parts of the signal and observing the change in the output.
Class-Activation Maps [98,99,100,101,107,113,114,115,116,117,118,119,120,121,122,123,125]	Local	Model-specific *[for CNN only]*	Post-hoc *[needs retraining]*	Highlights segments of an EG signal to indicate the contribution of each region by outputting the averaged and concatenated feature maps or by calculating the importance score through computing the gradients of the output class to the final convolutional layer.
Saliency Maps (SMs) [102,103,127,128]	Local	Model-agnostic *[for any NN]*	Post hoc	Suggests a segment of an ECG signal that contributes the most to classifying a particular input instance to an output class.
Learned internal parameters (LIPs) [94,95,96]	Global	Model-specific	Intrinsic	Provide the internal parameters of the ML models. For instance, the splitting conditions of the tree structure are based on functional feature components and provide the final decision probabilities on the leaf nodes.
Example-based (EB) [155]	Local	Model-agnostic	Post hoc	The explanation output consists of raw and combined information about ECG signals that are nearest neighbors to the ML model’s input ECG tracing.

**Table 7:** Attention mechanism-based–visual observation-based IML technique performance evaluation.

Method	Literature	Dataset	Disease Class	Remark
Attention Mechanism	Mousavi et al. [105]	MIT-BIH Atrial Fibrillation [49] 2017 PhysioNet Challenge [54,55]	Atrial Fibrillation (AF) Non-Atrial Fibrillation	The method highlights important heartbeats from an ECG signal.
	Jin et al. [106]	MIT-BIH Arrhythmia Database [48] China Physiological Signal Challenge [44]	Normal sinus rhythm (NSR) AF Premature Atrial Contraction (PAC) Premature Ventricular Contraction (PVC) Others	Authors claimed they made a comparison against the ground truth medical basis.
	Hong et al. [145]	2017 PhysioNet Challenge [54,55]	AF Non-Atrial Fibrillation	Authors showed the proposed explanation is less affected by noises.
	Yao et al. [146]	China Physiological Signal Challenge [44]	NSR, AF, I-AVB ^1^, LBBB ^2^, STE ^3^, RBBB ^4^, PAC, PVC, STD ^5^	A visual illustration was given only for PAV, PVC, and AF.
	Elul et al. [147]	Normal Sinus Rhythm RR Interval Database [52] Long-Term AF Database [168] MIT-BIH Atrial Fibrillation [49] MIT-BIH Arrhythmia Database [48] Telemetric and Holter ECG Warehouse [169] 2017 PhysioNet Challenge [54,55]	NSR, AF, LP-NSR ^6^, SVT ^7^, VT ^8^, Vent. trig. ^9^, Vent. big. ^10^, At. big. ^11^, Brady. ^12^, IR ^13^	The proposed model is compared against Grad-CAM both in terms visual explanation and quantitatively using attention scores.
	Mousavi et al. [148]	MIT-BIH Atrial Fibrillation [49]	Atrial Fibrillation (AF) Non-atrial fibrillation	The most important segments of an ECG are highlighted to give a visual explanation for the predicted output.

^1^ First-degree atrioventricular block, ^2^ Left bundle branch block, ^3^ ST-segment elevation, ^4^ Right bundle branch block, ^5^ ST-segment depression, ^6^ Latent pathology normal sinus rhythm, ^7^ Supraventricular tachycardia, ^8^ Ventricular tachycardia, ^9^ Ventricular bigeminy, ^10^ Ventricular trigeminy, ^11^ Atrial Bigeminy, ^12^ Bradycardia, ^13^ Idioventricular rhythm.

**Table 8:** Class activation maps based visual observation based IML techniques performance evaluation.

Method	Literature	Dataset	Disease Class	Remark
Class Activation Maps	Goodfellow et al. [114]	2017 PhysioNet Challenge [54,55]	NSR AF Other Rhythm	The CAM gives a visual presentation of segments of ECG signal that the ML model used more for making classification decision.
	Goswami et al. [113]	MIT-BIH Arrhythmia Database [48]	PVC Control [other beats]	CAM is used to reveal the prominent segments of the ECG signal in heuristically driven heartbeat level weakly supervised learning.
Gradient-based CAMs	Porum et al. [98]	MIT-BIH Normal Sinus Rhythm [50,55] BIDMC-Congestive Heart Failure Database [51]	Congestive Heart Failure (CHF) Control [normal beats]	Grad-CAM heat map based visualization of individual heartbeats contributed for CHF classification is implemented.
	Wang et al. [115]	MIT-BIH Arrhythmia Database [48] PTB-XL ECG dataset [46,58]	Normal (N) Supraventricular-ectopic beats (S) Fusion beats (F) Ventricular ectopic beats (V) Unknown beats (Q)	Grad-CAM is used to visualize regions of heartbeats contributed most for the classification.
	Raza et al. [116]	MIT-BIH Arrhythmia Database [48]	N, S, F, V, Q	Grad-CAM is used to visualize the contribution of beat segments in the classification output.
	Ganeshkumar et al. [117]	China Physiological Signal Challenge [44]	NSR, AF, I-AVB, LBBB, RBBB, PAC, PVC, STD, STE	Grad-CAM is used to visualize the contribution of ECG segments in the classification output.
	Jahmunah et al. [99]	PTB Diagnostic ECG Database [170]	Myocardial Infraction (MI) Control [normal beats]	Grad-CAM is used to visualize the contribution of ECG segments for MI classification.
	Lopes et al. [118]	Phospholamban (PLN) cardiomyopathy dataset [141]	Phospholamban Control (Non-phospholamban)	Important regions of an ECG that contributes the most to the model classification are visualized using Grad-CAM. The result showed QRS complex played a major role. However, other authors reported PLN detection is dependent on T-wave [141].
	Cho et al. [120]	12- and 6-lead ECG compiled by authors.	MI Control [non-MI]	Grad-CAM is used to highlight the ECG signal segments based on their contribution for final segmentation.
	Kwon et al. [121]	12-, 6-, and 1-lead ECG compiled by authors.	Cardiac arrest event Control [non-event]	A heatmap from Grad-CAM is used to visualize important regions of an ECG signal-based on their contribution to the model’s prediction.
	Lee and Shin [107]	2017 PhysioNet Challenge [54,55]	NSR, AF, other rhythm abnormalities, noisy	The article presented Grad-CAM localized regions on electrocardiomatrix (ECM) at the intermediate block of the model. However, the general interpretability of the overall technique is not simple to be understood by physicians, this is mainly, the signal domain transformation.
	Li et al. [119]	12-lead ECG compiled by authors.	NSR, AF, I-AVB, CRBBB ^1^, LAFB ^2^, PVC, PAC, ER ^3^, TWC ^4^	Grad-CAM heatmap is used to visually highlight the important segments of an ECG used for the classification. However, the explanation technique is not well experimented.
	Sangha et al. [122]	12- lead CODE dataset [18]	I-AVB, RBBB, LBBB, SB ^5^, AF, ST ^6^	A model trained with mage based ECG is explained using a Grad-CAM for properly classified 25 RBBB and LBBB cases.
	Kwon et al. [123]	12-lead ECG compiled by authors.	Aortic Stenosis Control [non-aortic stenosis]	A model trained with demographic information, hand-crafted ECG features and raw ECG signals. Grad-CAM is used to explain model’s prediction output through generating a heatmap with scale importance.
Guided Grad-CAM	Aufiero et al. [125]	12-lead ECG compiled by authors and not available publicly.	Congenital long QT syndrome (LQTS) NSR (Control)	Grad-CAM score is used to explain the component of an ECG signal that contributes most for LQTS detection. The Grad-CAM explanation score is obtained after experimenting on correctly classified test dataset.
Grad-CAM++	Fang et al. [101]	PTB-XL ECG dataset [46,58]	MI Control [Healthy]	A Grad-CAM++ is used to visualize an MI prediction model output of a 3-D ECG image.
	Jiang et al. [124]	China Physiological Signal Challenge [44]	NSR, AF, IAVB, LBBB, RBBB, PAC, PVC, STD, STE	Grad-CAM++ generates a heatmap that superimposed on an ECG signal to provide an visualize the contribution of various ECG segments.

^1^ Complete right bundle branch block, ^2^ Left anterior fascicular block, ^3^ Early repolarization, ^4^ T-wave change, ^5^ Sinus Bradycardia, ^6^ Sinus Tachycardia.

**Table 9:** Occlusion Maps, Saliency Maps, and LRP based Visual observation based IML techniques performance evaluation.

Method	Literature	Dataset	Disease Class	Remark
Occlusion Maps	Bodini et al. [102]	2020 PhysioNet Challenge [47,56]	PR ^1^, LQT ^2^, AF, AFL ^3^, LBBB, QAb ^4^, TAb ^5^, LPR ^6^, LQRSV ^7^, I-AVB, PAC, LAD ^8^, SB, Brady., NSR, ST, PVC, SA ^9^, LAFB, RAD ^10^, Tinv ^11^, NSIVCD ^12^, IRBBB ^13^, CRBBB	Relevance of three ECG signal components, i.e., P-wave, QRS complex, and T-wave computed after occlusion and the visual explanation shows the important regions of an ECG signal.
	Bleijendaal et al. [141]	PLN dataset collected by authors.	PLN cardiomyopathy Control (non-PLN)	Occlusion maps are generated through the setting-occluded segment of the ECG’s signal to zero. The visual result shows the most important regions of the ECG that the model used for identifying PLN. Furthermore, the technique was validated by an expert cardiologist and showed comparable results.
Saliency Maps	Bodini et al. [102]	2020 PhysioNet Challenge [47,56]	PR, LQT, AF, AFL, LBBB, QAb, TAb, LPR, LQRSV, I-AVB, PAC, LAD, SB, Brady., NSR, ST, PVC, SA, LAFB, RAD, Tinv, NSIVCD, IRBBB, CRBBB	The visual saliency maps with quantitative relevance values of each segment of an ECG is provided.
	Bridge et al. [103]	Authors claimed the scanned-ECG data are taken from Deng et al. [171] and not publicly available	NSR Abnormal rhythm	The visual explanation is provided by saliency map. However, the model is trained with a very limited scanned ECG image data.
	Kwon et al. [127]	Authors collected the dataset	Pulmonary hypertension (PH) Non-pulmonary hypertension	Saliency map is used to visually explain the regions of an ECG that contributes the most in the model’s classification output.
	Jo et al. [128]	Authors collected the dataset	NSR, AF, SVT, VT, PM ^14^, JR ^15^, CAVB ^16^, 2AVB-T2 ^17^, 2AVB-T1 ^18^	Saliency method is used to visually explain the regions of an ECG signal that contributes the most for detecting the ECG features such as AV sequencing.
Layer-wise relevance propagation (LRP)	Strodthoff et al. [104]	PTB-XL ECG dataset [46,58] China Physiological Signal Challenge [44]	PVC PACE	The proof of concept of LRP based visual explanation is provided only done for PVC and rhythm PACE.

^1^ Pacing rhythm, ^2^ Prolonged QT interval, ^3^ Atrial flutter, ^4^ Q-wave abnormal, ^5^ T-wave abnormal, ^6^ Prolonged PR interval, ^7^ Low QRS voltage, ^8^ Left axis deviation, ^9^ Sinus arrhythmia, ^10^ Right axis deviation, ^11^ T-wave inversion, ^12^ Nonspecific intraventricular conduction disorder, ^13^ Incomplete right bundle branch block, ^14^ Pacemaker rhythm, ^15^ Junctional rhythm, ^16^ Complete atrioventricular block, ^17^ Second-degree atrioventricular block Mobitz type II, ^18^ Second-degree atrioventricular block Mobitz type I.

**Table 10:** Analysis of the feature effects via SHAP, feature importance, and a LIME-based IML performance evaluation.

Method	Literature	Dataset	Disease Class	Remark
SHAP	Angelaki et al. [68]	Authors collected the dataset	Normal Geometry Left ventricular hypertrophy (LVH) Concentric remodeling (CR)	The SHAP ranked the global feature importance of an ECG signal and provided a local explanation for the model’s classification output.
	Rouhi et al. [69]	2017 PhysioNet Challenge [54,55]	AF Control group *[ NSR, Other Rhythm, Noisy recording]*	The authors did not evaluate the clarity and soundness of their proposed technique but showed the improvement SHAP techniques bring to the random forest classifier.
	Anand et al. [70]	PTB-XL ECG dataset [46,58]	CD, HYP, MI, NSR, STTC	The SHAP highlights the important morphological segments of an ECG signal to emphasize the features that lead the model to the particular classification output.
	Ibrahim et al. [71]	ECG-ViEW II [172]	Acute Myocardial Infraction (AMI) Control (not AMI)	The SHAP ranked the ECG signal features on their level of impact on the model output.
	Neves et al. [80]	MIT-BIH Arrhythmia Database [48]	N, S, F, V, Q	The SHAP identified morphological regions of an ECG signal to emphasize the features that contribute the most to the model to decide the classification output. In addition, to measure the interpretation performance, the authors used quantitative techniques.
	Al-Mahfuz et al. [111]	MIT-BIH Arrhythmia Database [48]	N, LBBB, RBBB, PVC, PB	The SHAP values showed the contribution of the ECG signal frequency components in output prediction using a time-frequency representation of the ECG signal.
	Wickrammsinghe and Athif [109]	China Physiological Signal Challenge [44]	PR, LQT, AF, AFL, QAb, TAb, LPR, LQRSV, I-AVB, LAD, SB, ST, SA, RAD, Brady., NSR, LAFB, Tinv, NSIVCD, IRBBB, BBB ^1^, PRWP ^2^, [CRBBB, RBBB], [CLBBB, LBBB], [PAC, SVPB ^3^], [PVC, VPB ^4^]	The SHAP values showed features around a segment of an ECG signal that dominates the classification output
	Zhang et al. [110]	China Physiological Signal Challenge [44]	NSR, IAVB, AF, LBBB, RBBB, PAC, PVC, STD, STE	The SHAPs the important morphological segments of an ECG signal to emphasize the features that lead the model to the particular classification output.
Feature-Importance	Neves et al. [80]	MIT-BIH Arrhythmia Database [48]	N, S, F, V, Q	The authors used PFI to measure the importance of a feature through perturbing it and witnessing the model’s output performance. The more important the feature, the higher the loss in performance
	Krasteva et al. [164]	2017 PhysioNet Challenge [54,55]	NSR, AF, Other arrhythmia, Noise	Authors identified influential features by their relative importance for the ML classification output.
	Hua et al. [165]	Hefei Hi-tech competition	NSR, AF, QRS low voltage, Short PR interval	Authors identified features that have meaningful clinical context.
LIME	Bodini et al. [81]	The PTB Diagnostic ECG Database [170]	STE-MI Healthy Control	A LIME is used to localize segments of an ECG signal that contributed most for the classification.
	Neves et al. [80]	MIT-BIH Arrhythmia Database [48]	N, S, F, V, Q	A local surrogate model is used to identify important features that contribute the most to the model’s output classification.

^1^ Bundle Branch Block, ^2^ Poor R wave progression, ^3^ Supraventricular Premature Beats, ^4^ Ventricular Premature Beats.

## Data Availability

Not applicable.
